# Association between adolescent obesity and early adulthood healthcare utilization—a two-cohort prospective study

**DOI:** 10.1186/s12916-025-03866-w

**Published:** 2025-01-21

**Authors:** Emilia Hagman, Vidar Halsteinli, Resthie R. Putri, Christina Hansen Edwards, Gudrun Waaler Bjørnelv, Claude Marcus, Rønnaug A. Ødegård

**Affiliations:** 1 Department of Clinical Science, Intervention, and Technology, Karolinska Institutet, Huddinge, Sweden; 2 Department of Surgery, Obesity Research Centre, St. Olavs Hospital Trondheim University Hospital, Trondheim, Norway; 3 Department of Public Health and Nursing, Norwegian University of Science and Technology, Trondheim, Norway; 4 Department of Child and Adolescent Health Promotion Services, Norwegian Institute of Public Health, Trondheim, Norway; 5 Division of Mental Health Care, St. Olavs Hospital, Trondheim University Hospital, Trondheim, Norway; 6 Department of Health Management and Health Economics, University of Oslo, Oslo, Norway; 7 Department of Clinical and Molecular Medicine, Norwegian University of Science and Technology, Trondheim, Norway

**Keywords:** Adolescent obesity, Healthcare Costs, Healthcare utilization, Health economics, Real-world data

## Abstract

**Background:**

Pediatric obesity is a growing global health challenge, with long-term implications for individuals and healthcare systems. Existing studies on the association between pediatric obesity and healthcare use in adulthood are limited and often rely on mathematical simulation models. This study aims to provide real-world data on the impact of adolescent obesity on specialized healthcare utilization and costs in early adulthood.

**Methods:**

This study analyzed data from two longitudinal cohorts: a population-based cohort from Norway (Young-HUNT) and a clinical cohort from Sweden (BORIS), the latter with matched general population comparators. Individuals included were born between 1987 and 1994, with BMI measurements at ages 13–19, and follow-up data from ages 20 to 30 years. Healthcare utilization and costs were assessed using national patient registries.

**Results:**

A total of 7592 individuals from Norway (5.7% with adolescent obesity) and 1543 individuals from Sweden with adolescent obesity, accompanied with 7330 matched general population comparators, were included. Among females, adolescent obesity was associated with significantly higher specialized healthcare utilization and costs in young adulthood, e.g., in Sweden, females with adolescent obesity had a 57% probability of annual specialized healthcare visits at ages 25–29, compared to 49% among the general population, *p* < 0.0001. In Norway, a similar pattern was observed. Among males, the association between obesity and healthcare utilization/annual specialized visits was less prominent. Annual excess costs for females with a history of adolescent obesity ranged from €578 to €835, while males showed minimal or no annual excess costs.

**Conclusions:**

Analyses of real-world data cohorts from Norway and Sweden reveal that adolescent obesity is associated with increased healthcare utilization and costs in young adulthood, exceeding previous estimates. A distinct sex difference was evident, with females incurring higher costs compared to males.

**Supplementary Information:**

The online version contains supplementary material available at 10.1186/s12916-025-03866-w.

## Background

Pediatric obesity is one of the most serious health challenges of the twenty-first century. If the increasing trends persist, more than 750 million children and adolescents are expected to be living with overweight or obesity in 2035 [1]. Over the life span, obesity is associated with cardiometabolic comorbidities as well as psychiatric conditions [2, 3]. Additionally, obesity increases the risk of several cancers, autoimmune diseases, and even premature mortality [3, 4]. Beyond the health consequences for individuals, obesity places a considerable burden on healthcare systems and society as a whole. Healthcare utilization related to obesity tends to peak from middle age onward, while a smaller increase is observed during early adulthood [5]. However, whether this pattern also holds for pediatric obesity is unclear. Both patterns of comorbidities and the genetics associated with pediatric obesity differ from adult-onset obesity [6]. Moreover, the longer duration of obesity in those affected from an early age may lead to increased healthcare utilization during early adulthood [7]. There is a growing body of evidence suggesting that pediatric obesity leads to increased utilization of healthcare services during childhood [8–17]. However, not all studies have detected increased healthcare utilization or costs [18–21]. The diversity in past findings may be due to substantial between-study heterogeneity, reflecting differences in healthcare systems, delivery of services, and data quality [17]. Moreover, studies of the association between pediatric obesity and healthcare use and costs in adulthood are limited and mainly based on mathematical simulation modelling approaches and not real-world individual longitudinal data [7, 22, 23]. While cost estimates from simulation models are useful for understanding long-term healthcare needs, models often depend on assumptions that may simplify the complexity causing unreliable estimations. Therefore, access to real-world data is crucial for improving the accuracy of these estimates and making informed decisions guiding the treatment and prevention of obesity and its comorbidities. In the current study, we used prospectively collected real-world individual-level data from two neighboring countries, Norway and Sweden, which share major similarities both with respect to population and health system characteristics. Our aim was to examine if adolescent obesity increases specialized healthcare utilization and associated costs in early adulthood.

## Methods

### Study design, data sources, and population

In this study, we analyze data from two dynamic cohorts: one from Norway and one from Sweden. Both countries’ cohorts are prospectively collected and are linked to individual-level data. The Norwegian cohort was based on a population survey and clinical measurements, while the Swedish cohort was a clinical sample that received obesity treatment during adolescence and a matched group from the general population.

#### Norwegian cohort

The third wave of the Trøndelag Health Study (Young-HUNT3) took place in 2006–2008 in the northern part of Trøndelag county in Norway (http://www.ntnu.edu/hunt). The area is mostly rural, with a lack of large cities, but is considered fairly representative of the Norwegian population with regard to geography, economy, sources of income, age distribution, morbidity, and mortality [24]. A total of 10,464 adolescents in lower and upper secondary schools were invited to answer public health-related questions. Of these, 74% (*n* = 7716) completed both the questionnaire and had anthropometric measurements [24]. Height and weight were measured objectively by trained nurses. At the time of the Young-HUNT3 survey, treatment for adolescent obesity was not systematically available in the county. All participants provided consent in accordance with Norwegian laws and recommendations. The present study was approved by the Regional Committee of Ethics in Medical Research (2016/537).

#### Swedish cohort

The Swedish Childhood Obesity Treatment Register (BORIS) (http://www.e-boris.se/in-english/) was initiated in 2005 and is a prospective register of children and adolescents undergoing obesity treatment in Sweden [25]. The register comprises data, including anthropometric measurements, from clinical visits. Pediatric obesity treatment in Sweden consists mainly of behavioral lifestyle therapy. However, bariatric surgery in adolescents has been performed in studies [26] and has become an option in recent years for selected adolescent patients. Glucagon-like-peptide-1 receptor analogues for the treatment of obesity were not approved for adolescents in Sweden during the time frame of the present study. Through the personal identity number assigned to all residents in Sweden, approximately 32,000 patients who were registered in BORIS in December 2020, were eligible for a linkage with national registers. A contemporaneous comparison group from the Total Population Register (held by Statistics Sweden) was matched with a ratio of 1:5 by sex, year of birth, and residential area (*n* ≈ 157,000). Height or weight measurements were not available in the comparison group. Ethical approval was obtained by the regional Ethics Committee in Stockholm, Sweden (No. 2016/922–31/1, 2020–02707).

### Study setting

Norway and Sweden share similarities in their healthcare systems providing unique opportunities for joint register-based research, but there are also differences [27]. In both countries, healthcare is provided within a public healthcare system with universal access. The major source of funding is taxes in both countries [28]. In Sweden, 21 counties have responsibility for providing somatic and psychiatric specialized healthcare and primary healthcare. In Norway, four Regional Health Authorities owned by the state are responsible for providing somatic and psychiatric specialized care. Unlike Sweden, 356 municipalities have the responsibility of providing primary healthcare in Norway.

Individual-leveled data from all visits to specialized healthcare is comprehensively collected by the administrative registers; the Norwegian Patient Register and the Swedish Patient Register respectively. However, neither register includes visits to primary care.

#### Study populations

**Fig. 1:**
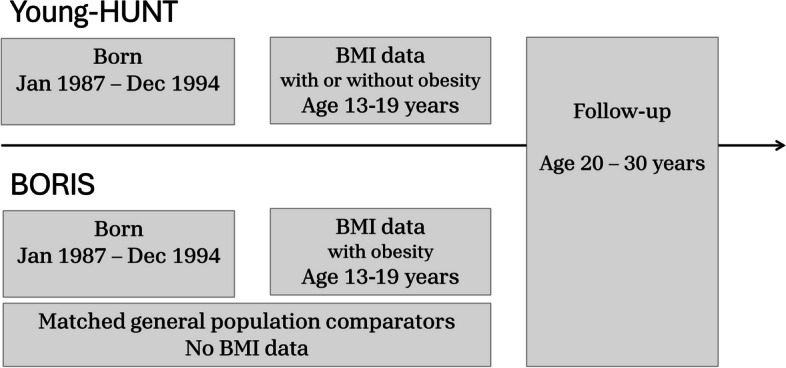
Study design. The Norwegian cohort consisted of the population-based survey Young-HUNT, and the Swedish cohort consisted of patients undergoing obesity treatment (BORIS) and general population comparators that were matched on sex, year of birth and residential area at the obesity treatment initiation for the BORIS-cohort

Both cohorts included individuals born from January 1, 1987, to December 31, 1994, with body mass index (BMI) data between age 13 and 19 years. The outcome was assessed from 20 years of age up to 30 years of age. The study design is illustrated in Fig. 1. To capture the regular adolescent obesity population, some exclusion criteria were applied in both cohorts: genetic syndromes (Morbus Down, Prader Willi, Laurence-Moon-Bardet-Biedl, Turner, Noonan, Klinefelter, Russel, and Fragile-X), weight loss surgery prior to start of follow-up, and secondary obesity due to craniopharyngioma. Additionally, in the Swedish dataset, individuals diagnosed with intellectual disabilities were excluded to ensure comparability with the Norwegian cohort, which was derived from a school-based sample. Moreover, individuals who emigrated or were deceased before the start of follow-up were excluded. These exclusion criteria could not be applied to the Norwegian data, as the dataset did not include this information. Process of inclusion and exclusion and associated International Statistical Classification of Diseases and Related Health Problems, Tenth Revision (ICD-10) codes are provided in Additional file 1: Table S1 and Table S2.

#### Register linkage procedure

In both countries, all residents are assigned a unique national personal identification number, which was used to link the cohorts to the administrative national medical registers, the Norwegian Patient Register and the Swedish Patient Register. In the current study, data on visits to specialized healthcare was used to assess healthcare utilization, diagnosis-related group (DRG) was used to estimate costs, and primary diagnoses were used to describe reasons for visits. This study utilized data available from the Norwegian Patient Register spanning January 2008 to December 2017 and from the Swedish Patient Register from January 2008 to July 2023. Given the dynamic cohorts, the number of individuals contributing with data decreased with older age, Additional file 1: Table S3. The Norwegian Patient Register was at the time held by the Norwegian Directorate of Health, and the Swedish Patient Register was and is held by the National Board of Health and Welfare.

### Variables and definitions

#### Exposure and confounders

The exposure, obesity in adolescence, was defined according to the International Obesity Task Force criteria [29]. Data on sex was obtained from survey data in Norway and from the Total Population Register in Sweden. Since socioeconomic position may affect both the prevalence of obesity and healthcare utilization, educational level was assessed and dichotomized based on completion of ≥ 12 years of schooling (corresponding to upper secondary school) at the age of 20. In Norway, data on education was obtained from Statistics Norway, and in Sweden, data was obtained from The Longitudinal Integration Database for Health Insurance and Labor Market Studies, held by Statistics Sweden.

### Outcomes

The following outcomes were applied: (1) the probability of any visit to specialized healthcare (specialized outpatient care or inpatient care) per year when individuals were between 20 and 30 years of age, (2) annual frequency of visits to specialized healthcare, both conditional and unconditional of any visit, and (3) annual cost of visits based on the summed cost of specialized healthcare. For cost calculations, cost-weights for DRGs were used, and for participants with no visit, costs were set at zero. Both the Norwegian and the Swedish DRGs are based on the grouping system NordDRG (https://nordcase.org). The exact number of DRGs differs over the years and between countries but typically consists of between 1000 and 2000 DRGs. Each DRG is assigned a cost-weight that represents the historical average cost of visits within the specific DRG across each country. We used the DRG assigned to each visit and calculated costs by multiplying the associated cost-weights by a national tariff corresponding to a weight of 1. Since mental health services and drug and alcohol treatment were not part of the DRG system in Norway for the study period, a national unit cost published by the Norwegian Directorate of Health to calculate the cost per outpatient visit was used. Similarly, for inpatient care, the length of stay was multiplied by a national per-day unit cost. All costs were inflation-adjusted to 2022 using the Norwegian consumer price index and the Swedish medical consumer price index and converted to euro (1 EUR = 10.1021 NOK = 10.6349 SEK in 2022).

Diagnostic information for visits was distributed across a wide range of ICD-10 codes. To provide clinically relevant descriptive data, conditions relevant to obesity were grouped by aggregating primary ICD-10 codes into six categories: cardiometabolic diseases, obesity, birth and pregnancy, psychiatry and substance abuse, musculoskeletal pain and injuries, malignant tumors, and a seventh category for the remaining ICD-10 codes. ICD-10 codes included in each category are described in Additional file 1: Table S4.

### Data management and statistical analyses

Each country prepared and analyzed its own dataset. To ensure that the analyses were aligned, the syntax for formal analyses was written in Sweden, initially tested on a synthetic dataset created in Norway, and then applied to the prepared datasets from each country.

Descriptive analyses are presented as median (quartile 1 [Q1], quartile 3 [Q3]) for continuous variables and as frequencies and proportions for categorical variables.

Analyses of the outcomes were stratified by sex and age category in adulthood (20–24 years and 25–29 years) and adjusted for attained educational level. Generalized estimating equations for panel data were applied to estimate all outcomes. To estimate the probability of having a visit, binomial distribution was used. To estimate the average number of visits and annual individual costs, Poisson distribution with log link was used [30]. In all models, robust standard errors were estimated to obtain 95% confidence intervals. Given that the correlations between each pair of observations within-person varied substantially, unstructured correlation was applied in all models, except when the average number of visits was the outcome. The latter assumed exchangeable correlation given the lack of convergence in the model when unstructured correlation was assumed. Missing data was handled with listwise deletion except for when costs were estimated, where missing values were set at 0.

Formal analyses of healthcare utilization were performed in Stata (Statacorp, College Station, TX, USA) version 17.0 in Norway and 16.1 in Sweden. SAS statistical software (version 9.4, Cary, NC) was used for data preparation and descriptive statistics in Sweden.

## Results

From Norway, 7592 individuals were included, of which 430 (5.7%) had obesity in adolescence. From Sweden, 1543 individuals with adolescent obesity and 7330 matched general population comparators were included. The proportion of males was 50.1% in the Norwegian cohort and 53.5% in the Swedish cohort. Of those with obesity, the median (Q1, Q3) adolescent BMI was 31.2 (29.9,33.6) in Norway and 33.7 (30.9, 37.2) kg/m^2^ in Sweden. Of those without obesity in Norway, 3.6% had underweight, 71.5% normal weight, and 19.3% overweight. This information was not available among the Swedish general population comparators. Since the Swedish obesity cohort was derived from a clinical sample, obesity treatment data was available. The median (Q1, Q3) treatment duration was 21.5 (8.3, 40.2) months, the number of treatment visits was 3 (2, 6), and the change in BMI was 0.00 (− 0.21, 0.12) kg/m^2^. Cohorts’ characteristics are provided in Table 1.

**Table 1:** Characteristics of study populations

	**Norway**		**Sweden**	
	**Ung-HUNT obesity *n* = 430**	**Ung-HUNT no obesity *n* = 7 162**	**BORIS (obesity) *n* = 1 543**	**Matched**^a^** general population comparators *n* = 7 330**
Males, *n* (%)	245 (57.0)	3 559 (49.7)	826 (53.5)	3 925 (53.5)
Adolescent age, median (Q1, Q3)	16.0 (14.6,17.3)	15.6 (14.3,17.2)	14.8 (13.2, 16.0)	Missing
Adolescent BMI (kg/m^2^), median (Q1, Q3)	31.2 (29.9,33.6)	21.2 (19.4,23.4)	33.7 (30.9, 37.2)	Missing
Adolescent BMI SDS, median (Q1, Q3)	2.55 (2.39,2.81)	0.55 (−0.01,1.14)	2.94 (2.67, 3.24)	Missing
Educational level at 20 years of age				
< 12 years, *n* (%)	117 (27.2)	1 174(16.4)	385 (25.0)	971 (13.2)
≥ 12 years, *n* (%)	312 (72.6)	5 981(83.5)	1 141 (73.9)	6 298 (85.9)
Missing, *n* (%)	1 (0.2)	8 (0.1)	17 (1.1)	61 (0.8)

^a^Swedish general population comparators were matched on sex, year of birth, and residential area

The total number of specialized healthcare visits that contributed to the analyses was 90,122 in the Norwegian cohort and 102,748 in the Swedish cohort, over 51,091 and 81,455 person-years respectively, Additional file 1: Table S5. Of these, 90.7% and 91.3% were outpatient visits for the Norwegian and Swedish cohorts, respectively. On average, 35.8% of the Norwegian cohort with obesity and 35.2% without obesity had at least one visit to specialized healthcare annually. The corresponding numbers for the Swedish cohort were 47.0% of the individuals with obesity and 37.1% of the matched general population. Healthcare visits covered a wide range of diagnoses in both countries as shown in Additional file 1: Table S5. For the six obesity-relevant categories, increased healthcare utilization related to adolescent obesity was most prominent in the category “psychiatry and substance abuse”, particularly among females. In addition, for this diagnosis category, we observed substantially higher proportions in the Norwegian cohort compared to the Swedish cohort, regardless of adolescent obesity status.

### Probability of visits to specialized healthcare

In Sweden, both males and females who had adolescent obesity had a higher annual probability of specialized healthcare visits in adulthood compared to their general population comparators (Table 2). For instance, among females who experienced obesity in adolescence, the probability (95% CI) of having at least one visit annually was 0.56 (0.54–0.58) versus 0.43 (0.42–0.44) among general population comparators for ages 20–24 and 0.57 (0.54–0.59) versus 0.49 (0.47–0.50) for ages 25–29 years. In Norway, a similar pattern was found only for females in the age group of 25–29 years (Table 2).

**Table 2:** Estimated annual probability and average frequency of visits to specialized healthcare and their 95% confidence intervals (CI). Analyses were stratified for sex and adjusted for educational level

	**Females 20–24 years**		**Females 25–29 years**		**Males 20–24 years**		**Males 25–29 years**	
**Probability of any visit**		*p*-value		*p*-value		*p*-value		*p*-value
Norway								
Adolescent obesity	0.46 (0.41–0.50)	0.103	0.60 (0.54–0.66)	< 0.0001	0.27 (0.23–0.30)	0.617	0.28 (0.24–0.33)	0.331
No Adolescent obesity	0.42 (0.41–0.43)		0.48 (0.47–0.49)		0.27 (0.27–0.28)		0.26 (0.25–0.27)	
Sweden								
BORIS (obesity)	0.56 (0.54–0.58)	< 0.0001	0.57 (0.54–0.59)	< 0.0001	0.37 (0.34–0.39)	< 0.0001	0.35 (0.33–0.38)	< 0.0001
General population comparators	0.43 (0.42–0.44)		0.49 (0.47–0.50)		0.30 (0.30–0.31)		0.29 (0.28–0.30)	
**Visit frequency conditional of visit**^**a**^								
Norway								
Adolescent obesity	6.41 (4.78–8.03)	0.020	5.35 (3.81–6.90)	0.418	3.86 (3.11–4.61)	0.437	3.16 (1.99–4.32)	0.184
No Adolescent obesity	4.45 (4.23–4.67)		4.69 (4.25–5.12)		3.55 (3.31–3.79)		4.03 (3.46–4.61)	
Sweden								
BORIS (obesity)	3.41 (3.18–3.63)	< 0.0001	4.13 (3.81–4.46)	< 0.0001	2.56 (2.39–2.74)	0.297	2.67 (2.48–2.86)	0.087
General population comparators	2.80 (2.66–2.93)		3.21 (3.08–3.34)		2.46 (2.36–2.55)		2.47 (2.35–2.59)	
**Visit frequency unconditional of visit**^**b**^								
Norway								
Adolescent obesity	3.42 (2.32–4.52)	0.026	3.60 (2.49–4.72)	0.095	1.18 (0.82–1.53)	0.955	1.17 (0.75–1.59)	0.431
No Adolescent obesity	2.15 (2.01–2.29)		2.63 (2.37–2.88)		1.19 (1.08–1.30)		1.36 (1.16–1.56)	
Sweden								
BORIS (obesity)	2.14 (1.94–2.34)	< 0.0001	2.63 (2.37–2.90)	< 0.0001	1.05 (0.94–1.16)	0.001	1.08 (0.97–1.20)	< 0.0001
General population comparators	1.36 (1.27–1.45)		1.77 (1.68–1.87)		0.84 (0.79–0.89)		0.85 (0.80–0.91)	

^a﻿^Frequency of visits was conditional of having any visit to specialized healthcare

^b﻿^Frequency of visits was unconditional of having any visit to specialized healthcare

The average number of visits to specialized healthcare, conditional on at least one visit occurred, was higher among females who experienced obesity during adolescence in both Sweden and Norway at ages 20–24 and in Sweden alone at ages 25–29. For example, among females with adolescent obesity in Norway, the mean (95% CI) number of visits was 6.41 (4.78–8.03), while those without adolescent obesity had 4.45 (4.23–4.67) visits, *p* = 0.02. Conversely, among males, the frequency of visits, conditional at least one visit occurred, did not differ significantly between those who had experienced obesity in adolescence and those who had not, neither in Sweden nor Norway. The average frequencies of visits, conditional of having a visit, are provided in Table 2.

To reflect the frequency of visits on a group level, the average number of visits, regardless of whether any visit occurred, was estimated. Compared to no adolescent obesity, females who experienced adolescent obesity had higher visit frequency in both countries at ages 20–24 and in Sweden alone at ages 25–29. Additionally, males with a history of adolescent obesity in Sweden exhibited a greater frequency of visits in adulthood compared to the general population. This pattern was not observed in Norway. The estimated number of visits, unconditionally of any visit to specialized healthcare, is provided in Table 2.

### Costs

**Fig. 2:**
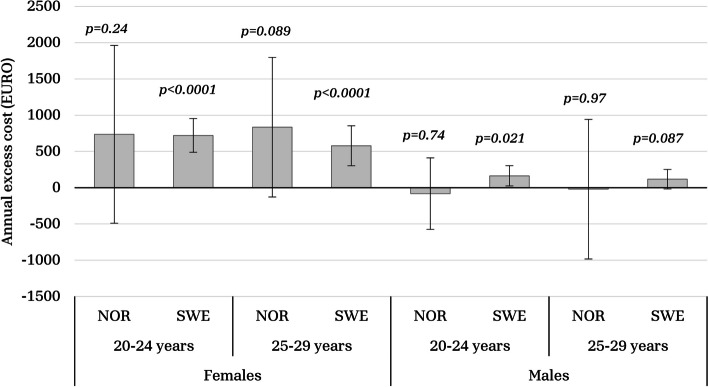
Average excessive annual cost for specialized healthcare for adolescent obesity in Norway and Sweden, respectively. Error bars represent 95% confidence intervals

Excess annual costs are illustrated in Fig. 2. In the Swedish cohort, female adolescent obesity was associated with an average (95% CI) annual excess cost of €721 (488–954) at 20–24 years of age and €578 (303–853) at 25–29 years of age. The estimates in the Norwegian cohort were similar, but not statistically significant; average annual excess cost of €736 (−490–1963) at 20–24 years of age and €835 (−128–1797) at 25–29 years of age. Among males with adolescent obesity, a modest incremental annual cost of €164 (24–304) was observed in the Swedish cohort for ages 20–24. No differences were found for the remaining male groups. Mean annual cost of specialized healthcare was in general higher for females than for males and in general higher in Norway than in Sweden (Table 3).

**Table 3:** Estimated average annual cost and 95% confidence interval (CI) for specialized healthcare, adjusted for educational level, among females and males, by age group country and adolescent obesity status. Costs are expressed in 2022’s Euro

Age group	Country	Adolescent obesity status	Cost (95% CI) among females Euro	Cost (95% CI) among males Euro
20–24 years	NOR	Obesity	2409 (1160–3658)	855 (416–1293)
		No obesity	1646 (1405–1887)	933 (741–1124)
	SWE	BORIS (obesity)	1576 (1354–1798)	666 (542–791)
		General population comparators	845 (767–924)	504 (444–564)
25–29 years	NOR	Obesity	2800 (1872–3729)	1195 (423–1967)
		No obesity	1904 (1437–2372)	1215 (631–1800)
	SWE	BORIS (obesity)	1790 (1527–2053)	682 (570–794)
		General population comparators	1204 (1112–1296)	566 (495–637)

## Discussion

This two-cohort study found that adolescent obesity was associated with increased specialized healthcare utilization in early adulthood not only in females both in Sweden and Norway but also among males in the Swedish cohort. This increase in healthcare utilization was reflected in higher costs for females with a history of adolescent obesity, while males with a history of adolescent obesity showed little to no excess healthcare costs in early adulthood.

As obesity rates continue to rise, so will the associated costs [31]. The largest costs of obesity and its consequences are likely to be indirect costs such as productivity loss and premature mortality, while direct costs [32], including medical expenses, are estimated to account for 8–31% of total costs in the WHO regions [31]. The present study focuses on direct costs, specifically in terms of specialized healthcare expenses. We found that the annual excess cost in early adulthood was substantially higher for females who experienced adolescent obesity. For males, no or only a slight excess cost was observed until 30 years of age. This pattern is in line with the previous estimation published by Sontag et al. [7]. However, the present study’s estimates from real-world data are higher compared to their simulation-based estimates. In adults, previous studies have investigated the causal relationship between BMI and healthcare costs using different methodological approaches [33–36]. These studies typically report a stronger association between BMI and healthcare costs when applying causal inference methods compared to traditional approaches. Similarly, a causal relationship between childhood obesity and healthcare utilization during childhood has been demonstrated [37]. However, the impact of childhood and adolescent obesity on healthcare utilization and costs in adulthood, particularly through causal inference methods, remains underexplored.

In the Norwegian cohort, males who experienced obesity in adolescence had numerically similar costs as their counterparts without adolescent obesity. The observed moderate excess cost for males with adolescent obesity in Sweden, but not in Norway, may be attributed to the Swedish cohort being derived from a clinical population. Individuals with obesity in the Swedish cohort may have had greater familiarity with healthcare services and thus have a higher propensity to seek medical care. Additionally, the degree of obesity in adolescence was greater in the Swedish cohort than in the Norwegian cohort (BMI 34 vs 31 kg/m^2^), which may contribute to increased disease severity. Among females in the present study, the annual excess cost of specialized healthcare associated with obesity ranged between €578 and €835, more than doubling some previous estimates [7, 22]. And in addition, the present estimates are higher than estimates in a review from 2018, where Hamilton et al. summarized total lifetime excess direct cost for girls with obesity to be €19 363 (2014 Euro) [22].

Among adults, obesity-related complications, such as type 2 diabetes and hypertension, have been suggested to be driving the medical costs associated with obesity [38]. In the current study, the proportion of all visits attributed to cardiometabolic diseases was relatively low (< 5% of all visits, Additional file 1: Table S5). This may be due to the age range investigated since several of the cardiometabolic diseases generally become apparent later in life. Additionally, conditions such as hypertension are typically managed in primary care, which was not included in the current study’s assessment. Also, psychiatric conditions have been shown to be more prevalent among people living with obesity, and evidence implies shared biological mechanisms between obesity and depression and anxiety [39, 40]. In the current study, visits attributed to psychiatric conditions and substance abuse accounted for over 40% of all visits in the Norwegian cohort, regardless of obesity in adolescence (Additional file 1: Table S5). This proportion was substantially lower in Sweden, ranging between 15% among females in the general population to 24% among males with adolescent obesity. The overall high proportion of visits due to psychiatric conditions and drug use disorders may mirror that these conditions are prevalent in the age range included in the present study [41]. The differences between Norway and Sweden are more intriguing. Even if the primary care is the first-line of care for psychiatric conditions in both countries, a larger proportion may have been referred to specialized care in Norway due to differences in the healthcare systems including disparities in access to specialized healthcare or even broader differences in access to psychiatric services overall.

Cross-country comparisons can provide valuable insights into health outcomes, costs, and health system performance, informing policy planning and supporting the transferability of interventions. However, these comparisons must be interpreted with caution, taking into account contextual factors such as differences in health system and clinical practice patterns [42], as well as methodological factors related to the outcome metrics applied [43]. Additionally, factors that may influence cost estimates, such as national differences in DRG systems and wage levels may affect cross-country comparisons [44]. In the present study, the two cohorts identified adolescent obesity in different settings; in Sweden, individuals were identified from a clinical cohort, while in Norway, individuals were identified from a population-based survey. Similarly, the control groups were sampled differently, where the Swedish group was derived from the general population (including individuals with obesity) while the control group in Norway was those in the population-based survey without obesity. Several actions were taken to ensure that the study design, data preparation, procedures, and formal analyses were aligned across both countries in the present study. Despite the above-mentioned complexities, we observed similarities in the patterns of healthcare use and costs in early adulthood associated with adolescent obesity which reinforces the generalizability of the results to countries with similar health systems.

### Limitations

Despite the real-world individual longitudinal data, there are some limitations to acknowledge. Firstly, only specialized outpatient care and inpatient care were assessed. Primary healthcare costs and costs for medical drugs were not available. Secondly, no weight trajectories were modeled, and consequently, we do not consider how obesity influences healthcare use and costs differently if obesity persists into adulthood or not. However, it is well-documented in the literature that adolescent obesity is challenging to treat and often persists into adulthood [45, 46]. This also was mirrored in the Swedish cohort of clinical obesity patients, where the average change in the degree of obesity was negligible. Additionally, no BMI data in the Swedish general population comparators were available. Thirdly, there are several factors that affect healthcare utilization which we have not taken into consideration, such as health inequalities. Adolescent obesity is associated with persistent or widened socioeconomic disadvantage in adulthood [47], which also have been linked to altered health and healthcare utilization [48]. In an attempt to address this, we adjusted for years of schooling. Further, weight discrimination and stigma may affect the healthcare of individuals living with obesity [47]. Additionally, since the Swedish and Norwegian patient registers include visits to a healthcare professional that may diagnose a patient (primarily medical doctors), visits to other healthcare professionals are to some extent not recorded. Hence, the total use of healthcare services in the present study is most likely underestimated.

Given that weight status may change during childhood, future research should evaluate the potential short- and long-term economic benefits of treating pediatric obesity. This is crucial for optimizing preventive services, weight management strategies, and understanding the long-term impact on healthcare spending.

## Conclusions

Analyses of real-world data cohorts from Norway and Sweden reveal that adolescent obesity is associated with increased healthcare utilization and costs in young adulthood, exceeding previous estimates. A distinct sex difference was evident, with females incurring higher costs compared to males.

## Supplementary Information

Additional file 1: Tables S1-S5. Table S1- Process of inclusion and exclusion. Table S2 – ICD-10 codes used at exclusion. Table S3 – Number of individuals with follow-up data by age. Table S4 – Categorization and description of reason for visit to specialized outpatient care and inpatient care. Table S5 – Reason for visits to specialized outpatient care and inpatient care.

## Data Availability

The data that support the findings of this study contains sensitive information. Restrictions therefore apply to the availability of these data, which were used under license for the current study, and so are not publicly available. According to Swedish law, Norwegian law and the General Data Protection Regulation, the authors are not permitted to share the datasets used in this study with third parties.

## Abbreviations

CI: Confidence interval
BMI: Body mass index
BORIS: The Swedish Childhood Obesity Treatment Register
DRG: Diagnosis-related groups
HUNT: The Trøndelag Health Study
ICD-10: International Statistical Classification of Diseases and Related Health Problems, Tenth Revision
Q1: Quartile 1
Q3: Quartile 3
