# Supplementary material for: Association between adolescent obesity and early adulthood healthcare utilization—a two-cohort prospective study
Source: BMC Med. 2025 Jan 21;23:33. doi: 10.1186/s12916-025-03866-w (PMC11752954; doi:10.1186/s12916-025-03866-w)
Supplement: Supplementary file 1 — Additional file 1: Tables S1-S5. Table S1- Process of inclusion and exclusion. Table S2 – ICD-10 codes used at exclusion. Table S3 – Number of individuals with follow-up data by age. Table S4 – Categorization and description of reason for visit to specialized outpatient care and inpatient care. Table S5 – Reason for visits to specialized outpatient care and inpatient care. [file 12916_2025_3866_MOESM1_ESM.pdf]

Additional file 1

Association between adolescent obesity and early adulthood healthcare utilization – a two-cohort prospective study

Emilia Hagman, Vidar Halsteinli, Resthie R Putri, Christina Edwards, Gudrun Waaler Bjørnelv, Claude Marcus, Rønnaug A Ødegård

Contents

Table S1. Process of inclusion and exclusion 2

Table S2. ICD-10 codes used at exclusion 3

Table S3. Number of individuals with follow-up data by age 4

Table S4. Categorization and description of reason for visit to specialized outpatient care and inpatient care 5

Table S5. Reason for visits to specialized outpatient care and inpatient care. 7

Table S1. Process of inclusion and exclusion

	Young-HUNT 3 Population-based cohort	BORIS register Clinical obesity treatment register	General population comparators to BORIS
Source	7 716[1]	32 184	
Born 1 Jan 1987 – 31 Dec 1994	7 716	2 315	7 594[2]
Obesity treatment initiation between 10-19 years of age		2109	
Obesity (IOTF) at 13 – 19 years of age		1 843	
Excluded:			
Missing height or weight data in adolescence	119	0	
Intellectual disability, syndrome, craniopharyngioma	1	134	5
Gastric bypass before 20 years of age	4	139	114
Emigration or death before 21 years of age	N/A	27	145
Number of individuals included	7 592		7 330
Obesity (IOTF) at 13 – 19 years of age (included)	430	1 543[2]	

[1]Participated in Young-HUNT 3
[2]Matched general population comparators were based on 1543 individuals from BORIS

Table S2. ICD-10 codes used at exclusion

Diagnosis/Procedure	ICD-10 code	Procedure Code
Intelectual diasability	F70-F79	
Down syndrome	Q90	
Prader-Willi syndrome	Q871	
Laurence-Moon-Biedi syndrome	Q878	
Russel-Silver syndrome	Q871G	
Noonan syndrome	Q871E	
Klinefelter’s syndrome	Q98	
Fragile X chromosome	Q992	
Turner syndrom	Q96	
Bariatric syrgery	E66	JDF[1]

[1]Surgical volume-reducing intervention on the ventricle

Table S3. Number of individuals with follow-up data by age

	Age in adulthood										Total person-time (years)
	20	21	22	23	24	25	26	27	28	29	
Norway											
Adolescent obesity	424	430	430	430	404	331	256	174	89	30	2 998
No adolescent obesity	7 117	7 162	7 162	7 162	6 560	5 120	3 713	2 407	1 294	396	48 093
Sweden											
BORIS	1 543	1 538	1 535	1 528	1 522	1 519	1 516	1 510	1 176	877	14 264
General population comparators	7 330	7 298	7 258	7 219	7 185	7 142	7 099	7 078	5 505	4 077	67 191

Table S4. Categorization and description of reason for visit to specialized outpatient care and inpatient care

Group and ICD-10 code(s)	Description
Cardiometabolic disease	
E10-E14	Diabetes mellitus
E28	Ovarian dysfunction
E78	Disorders of lipoprotein metabolism and other lipidaemias
K70-K85	Diseases of liver *and* Disorders of gallbladder, biliary tract and pancreas *excluding* Other diseases of pancreas and Disorders of gallbladder, biliary tract and pancreas in diseases classified elsewhere
I10-99	Hypertensive diseases *and* Ischaemic heart diseases *and* Pulmonary heart disease and diseases of pulmonary circulation *and* Other forms of heart disease and Cerebrovascular diseases *and* Diseases of arteries, arterioles and capillaries *and* Diseases of veins, lymphatic vessels and lymph nodes, not elsewhere classified *and* Other and unspecified disorders of the circulatory system
Obesity	
E65-E68	Obesity and other hyperalimentation
Birth and pregancy	
Z31-Z39	Procreative management *and* Pregnancy examination and test *and* Pregnant state, incidental *and* Supervision of normal pregnancy *and* Supervision of high-risk pregnancy *and* Antenatal screening *and* Outcome of delivery *and* Liveborn infants according to place of birth *and* Postpartum care and examination
O00-O99	Pregnancy, childbirth and the puerperium
Psychiatry and substance abuse	
F00-F69	Organic, including symptomatic, mental disorders *and* Mental and behavioral disorders due to psychoactive substance use *and* Schizophrenia, schizotypal and delusional disorders *and* Mood [affective] disorders *and* Neurotic, stress-related and somatoform disorders *and* Behavioral syndromes associated with physiological disturbances and physical factors *and* Disorders of adult personality and behavior
F90-F99	Behavioural and emotional disorders with onset usually occurring in childhood and adolescence *and* Unspecified mental disorder
Z00.4	General psychiatric examination, not elsewhere classified
Z03.2	Observation for suspected mental and behavioral disorders

F55-Z65	Persons with potential health hazards related to socioeconomic and psychosocial circumstances
**Musceloskelatal pain or injuries**	
M00-M99	Diseases of the musculoskeletal system and connective tissue
S00-S99, T00-T98	Injury, poisoning and certain other consequences of external causes
T00-T98	T00-T98
**Malignant tumors**	
C00-C97	Malignant neoplasms
**Others**	
All ICD codes not classified above	

Table S5. Reason for visits to specialized outpatient care and inpatient care

	Norway: Adolescent obesity			Norway: No adolescent obesity			Sweden: Adolescent obesity			Sweden: General population comparators		
**Women**	Individuals (n)	Visits (n)	% of all visits	Individuals (n)	Visits (n)	% of all visits	Individuals (n)	Visits (n)	% of all visits	Individuals (n)	Visits (n)	% of all visits
Cardiometabolic diseases	31	67	1.4	243	886	1.7	194	711	4.3	330	1 054	2.2
Obesity	36	223	4.6	26	118	0.2	244	798	4.9	47	109	0.2
Birth and pregnancy	74	719	14.9	1 363	10 189	19.1	298	2 402	14.7	1 534	8 859	18.7
Psychiatry and substance abuse	58	2323	48.1	830	19 455	36.4	293	3 293	20.1	1 016	7 179	15.2
Musculoskeletal pain and injuries	89	284	5.9	1 237	3 758	7.0	370	1 380	8.4	1 313	4 937	10.4
Malignant tumors	0	0	0.0	26	215	0.4	<10	76	0.5	21	103	0.2
Remaining diagnoses	147	1 211	25.1	2 642	18 802	35.2	654	7 729	47.2	2 872	25 036	53.0
In total		4 827	100.0		53 423	100.0		16 389	100.0		47 277	100.0
**Men**												
Cardiometabolic diseases	14	67	3.1	163	779	2.6	76	273	3.2	199	838	2.7
Obesity	14	54	2.5	<10	10	0.0	146	420	4.9	14	31	0.1
Birth and pregnancy	0	0	0.0	14	22	0.1	<10	16	0.2	38	80	0.3
Psychiatry and substance abuse	39	991	45.6	469	12 579	42.4	240	2 054	24.0	691	6 276	20.6
Musculoskeletal pain and injuries	73	337	15.5	102	4 224	14.2	378	1 378	16.1	1 903	7 336	24.0
Malignant tumors	<10	45	2.1	18	170	0.6	<10	72	0.8	17	161	0.5
Remaining diagnoses	136	680	31.3	2 046	11 914	40.1	621	4 337	50.7	2 732	15 810	51.8
In total		2 174	100.0		29 698	100.0		8 550	100.0		30 532	100.0

If less than 10 individuals contributed with data in a group, numbers of individuals are denoted to “<10” due to ethical reasons.
